# Functional neurological disorder, suggestion and virtual reality: neuroanatomical and computational perspectives

**DOI:** 10.1098/rstb.2024.0467

**Published:** 2026-09-17

**Authors:** Quinton Deeley

**Affiliations:** Cultural and Social Neuroscience Research Group, King's College London, London, UK

**Keywords:** functional neurological disorder, suggestion, hypnosis, brain, hierarchical predictive coding, attractor dynamics, virtual reality, cortical hierarchy

## Abstract

This Opinion piece addresses links between functional neurological disorder, hypnosis and suggestion, and their potential modulation by virtual reality (VR) in neuroanatomical and computational terms. The cortical hierarchy provides an anatomical basis for differing neurocognitive and computational theories applied to suggested effects and functional symptoms—hierarchical predictive coding, top-down cortical feedback and signal routing, and attractor dynamics. Each theory views the brain as a graded anatomical hierarchy with bidirectional influences between levels. Each considers that representations privileged by heightened attention (whether by cognitive control or automatic processes) alter the pre-conscious ‘editing’ of experience to generate suggested effects and functional symptoms. This occurs through an increase in top-down signals relative to bottom-up signals across the cortical hierarchy, resulting in enhanced activation of selected neuronal populations in intermediate levels. This alters the interpretation of bottom-up signals or radically misrepresents the presence or absence of bottom-up signals to higher levels. By contrast, VR influences experience by simulating the bottom-up effects of veridical sensory input to sensory processing streams. Despite the shared framework of the cortical hierarchy, the theories differ in important respects. Implications for future research and combining suggestions and VR to treat functional symptoms are considered.

This article is part of the discussion meeting issue ‘Digital healthcare for the management of functional neurological disorders’.

## 1. Introduction

Functional neurological disorder (FND) involves the presence of neurological symptoms that cannot be explained by neurological disease [1]. FND has long been linked to hypnosis and suggestion, both of which have been used as models for the pathogenesis of functional symptoms and as treatments [2,3]. More recently, virtual reality (VR) has been considered both as a potential treatment for FND, as a way of augmenting hypnosis and suggested effects, and—potentially—as a way of enhancing the effectiveness of hypnosis and suggestion in treating FND [4,5]. This raises the question of how functional neurological symptoms, suggested effects and VR are implemented in the brain and how they might interact.

Contemporary aetiological models of FND emphasise the role of belief and expectation in symptom formation and maintenance. For example,

FND is a disorder of sensorimotor processing in which erroneous health beliefs or expectations distort an often noxious somatosensory experience. This process is facilitated by misdirected and overly precise attention, anxiety and dissociation. The symptom formation helps ‘make sense’ of the amorphous somatic experience. The patient can be either consciously or pre-consciously complicit in it [6, p. 172].

Suggestions in hypnosis form part of the broader domain of suggestion, understood as ‘a form or type of communicable belief capable of producing and modifying experiences, thoughts and actions. Suggestions can be (a) intentional/non-intentional, (b) verbal/nonverbal, or (c) hypnotic/non-hypnotic’ [7, p. 111]. Specifically, hypnosis has been defined as an interaction between one person, the ‘hypnotist’, and another person or other people, the ‘subject’ or ‘subjects’. In this interaction, the hypnotist attempts to influence the subjects’ perceptions, feelings, thinking and behaviour by asking them to concentrate on ideas and images that may evoke the intended effects. The verbal communications that the hypnotist uses to achieve these effects are termed ‘suggestions’ [8]. Like functional neurological symptoms, suggested effects are subjectively realistic, and in the case of motor symptoms both are experienced as involuntary despite efforts to overcome them [9].

The influence of beliefs and expectations on suggested effects and functional symptoms can be contrasted with the experiential effects of VR, which simulates ‘real-world’ sensory inputs to evoke a subjectively realistic experience. These poles of influence are not independent. For example, hypnotic suggestibility enhances the sense of ‘presence’ or experiential immersion in VR [5]. Here we consider how the concept of the cortical hierarchy provides an anatomical framework for understanding how high-level representations such as beliefs, expectations and imagery influence brain function by ‘top-down’ effects, whereas VR influences brain function by simulating the ‘bottom-up’ effects of veridical sensory input to sensory processing streams. Evolving understandings of the cortical hierarchy support differing neurocognitive and computational models of functional symptoms and the effects of suggestion and VR, and how they might interact. We consider similarities and differences among these models, and implications for future research and treatment. The focus is on understanding suggested effects and functional symptoms in healthcare settings, noting that this explanatory approach can in principle be extended to understanding the effects of beliefs and expectations on normative variations in experience across diverse cultural settings.

## 2. The cortical hierarchy

A key proposal from the early days of neuroscience is that the cortex is organized into a graded anatomical hierarchy linking primary sensory cortices to progressively ‘higher’ association cortices [10,11]. Higher cortical levels, in turn, send projections to motor areas to implement goal-directed movements. Successive levels are connected through a ‘core synaptic hierarchy’, linking the axonal projections of neurons at one cortical level to dendrites of neurons at the next [11]. This ‘synaptic hierarchy’ is the connectional basis of the ‘cortical hierarchy’. Connections between cortical regions across the synaptic hierarchy are typically bidirectional, involving multiple feedforward and feedback projections, in addition to lateral projections to neurons at the same cortical level [10–13].

The cortical pathways of axonal projections originating in primary sensory cortices and connecting to higher-order cortical areas over successive synaptic steps are termed ‘feedforward’, ‘bottom-up’ or ‘sensory-fugal’ (from Latin *fugare*, to flee) [11]. These pathways were the first to be described in detail following landmark studies [12–14]. Feedforward pathways enable the conversion of basic sensory inputs into unified sensory percepts and—after higher cortical processing—motor commands.

In contrast to feedforward pathways linking successive cortical levels, lateral connections are either within the same cortical area as the parent neuron (intrinsic) or between cortical areas at a given level in the same or opposite hemispheres (extrinsic) [15]. Their diverse roles in integrating and augmenting information processing are considered below.

Detailed tract-tracing studies in the 1990s also delineated cortical projections which allow higher cortical levels to exert feedback influences on lower levels of processing at each cortical step [16,17]. These connections are described as ‘sensory-petal’ (from Latin *petere*, to seek), ‘top-down’ or ‘backward’ connections. There are more feedback than feedforward pathways, suggesting that feedback projections play a critical role in cortical function [18]. Cortical feedback pathways originate with pyramidal neurons, which project glutamatergic axons that depolarise their postsynaptic targets. Feedback axons synapse onto pyramidal neurons and GABAergic interneurons, although many of their cellular targets remain unknown [19].

There is, therefore, massive direct and indirect connectivity between cortical regions, levels and hemispheres. This connectivity allows similar sensory input to be linked to a wide range of cognitive and sensory–motor associations, and accompanying experiences [11,17,19]. These varied outcomes depend critically on feedback modulation. While feedforward pathways link sensation to cognition, feedback pathways implement goals represented at higher cortical levels, reflecting the appraisal of meaning and context based on prior learning [11,17,19].

Understanding the role of top-down modulation in suggested effects and functional symptoms, and their influence by VR, requires an account of how context and goals are represented at higher cortical levels, how they are influenced by prior learning and how their effects on signal processing at lower levels occur via feedback projections. To examine these multi-level processes we will consider the anatomy of the cortical hierarchy in more detail.

### (a) Laminar and cytoarchitectonic composition of the cortical hierarchy

Cortical areas at different levels of the cortical hierarchy were initially distinguished by architectonic patterns involving differences in cellular structure, the organization of neurons into laminae or layers, and myelination [20]. These architectonic tissue types are also called cortical types [21]. Microscopy revealed a spatially ordered gradient of successive changes from highly differentiated granular cortex with six clearly distinguishable layers of neurons and high myelin content, to less differentiated agranular cortex with four or fewer layers, lower neuron density and less myelin content [11,20].

This graded anatomical progression can be understood by visualizing the cortex as unfolded into a circular sheet with concentric rings of tissue types [11]. The outermost cortical ring is occupied by the primary sensory cortices for vision, hearing and touch, which themselves receive axonal projections from their respective sensory receptors via thalamic relay nuclei [11,22]. This outermost cortical ring is formed from koniocortex, the most clearly differentiated and specialised of the cortical zones containing densely packed small neurons in six distinct layers, a broad layer-IV and a high myelin content [22,23].

Each successive step is associated at a structural level with a reduction in differentiation of cortex (i.e. the layers are less clearly distinguishable) and at a functional level with increasing association and abstraction of information [11, 22–24]. In the case of visual or auditory processing, for example, primary sensory cortices are dominated by neurons which encode single stimulus features or categories [11,25]. Primary sensory cortical neurons project to upstream regions of unimodal association areas which encode basic features of sensation within a single sensory modality such as colour, motion, shape or pitch. In turn, these neurons send projections to neurons in downstream unimodal cortices which encode more complex sensory features such as objects, faces, word-forms, spatial locations or sound sequences [11]. Upstream and downstream unimodal association cortices are formed from eulaminate III and II cortices, which retain six layers but with a gradient of reduced differentiation [11,21].

Subsequent synaptic levels are occupied by heteromodal, paralimbic and limbic cortices. Heteromodal cortex is still formed from six layers—eulaminate I—but with less differentiation. The dysgranular layer of paralimbic cortex has only an incipient layer-IV, while the agranular limbic cortex lacks layer-IV completely [11]. Together, they are known as *transmodal* association cortices because they link information from different sensory modalities into multi-modal representations. They have a high proportion of mixed selectivity neurons that encode combinations of stimulus features and contextual variables [26]. As Mesulam put it, transmodal association cortices ‘provide critical gateways for transforming perception into recognition, word-forms into meaning, scenes and events into experiences, and spatial locations as targets for exploration’ [11, p. 1013].

Brain regions dominated by heteromodal cortex include regions in the prefrontal cortex (PFC), posterior parietal cortex, lateral temporal cortex and parahippocampal gyrus. Paralimbic cortex forms parts of the caudal orbitofrontal cortex, insula, the temporal pole and parahippocampal gyrus complex [11]. The limbic cortices of the amygdala and hippocampus, the innermost level of the ring and most remote from primary sensory cortices in terms of synaptic steps, have dense reciprocal connections with the hypothalamus. This allows sensory stimuli to be linked to endocrine and metabolic changes in the internal milieu in response to their perceived significance. Regions of association cortices also send axonal projections to motor areas to implement movements. The primary motor cortex is eulaminate with large layer-V pyramidal neurons (Betz cells) [11].

A forward processing hierarchy is preserved for the other sensory modalities of taste, smell and interoception, but with variations such as the number of processing steps, cortical targets and links to the hypothalamus [25]. Hierarchical forward processing can be illustrated by tracing information processing within the visual modality up to the level of ‘object invariance’—a stable visual percept constructed from the basic features of visual information coded in V1 [25]. Visual perception in the ventral ‘what’ pathway involves a hierarchically organized series of four to five stages of feedforward processing (V1→V2→V4→IT), transforming simple spatial filtering into discrete object representations [25]. Processing to the visual object level is mainly unimodal—in other words, operating on visual information alone. However, these percepts are then available for further processing in transmodal association cortices to determine their meaning, emotional significance and relevance for behaviour [11,17].

### (b) From regions to networks

Sensory processing streams eventually project to transmodal association cortices. In his influential paper ‘From Sensation to Cognition’ [11], Mesulam identified transmodal regions—‘nodes’, ‘epicentres’ or ‘hubs’—that perform associative transformation of incoming sensory information relevant to their neuropsychological domain. Mesulam identified at least five such epicentres—the network for spatial awareness involving the posterior parietal cortex and frontal eye fields (FEFs), Wernicke and Broca's areas for the language network, the hippocampal–entorhinal complex and the amygdala for the explicit memory/emotion network, the midtemporal and temporopolar cortices for the face-object recognition network and the lateral PFC and posterior parietal cortex for the working memory-executive function network.

Subsequent research showed that while these regions are involved in these neuropsychological domains, they form part of more extended neural networks supporting large-scale cognitive functions. An influential resting state connectivity study in 1000 healthy adults identified somatomotor and visual networks displaying largely local connectivity [27]. A more extended sensory-motor pathway forming the dorsal attention network, which guides eye movements based on attentional priority, showed stepwise hierarchical organization. However, seed regions placed in parietal and prefrontal association cortices revealed distributed but interdigitated large-scale networks of association cortex. While these have been named as the default network, control network and salience network, their roles in cognition are complex and continue to be defined. Each network links regions spanning multiple association regions (prefrontal, temporal, parietal association cortices and the cingulate) and lacks strong coupling to sensory or motor areas. Task-based functional magnetic resonance imaging (fMRI) reveals similar co-activation of widely distributed networks across association cortices [28].

### (c) Canonical and non-canonical circuits

These fMRI studies, along with increasingly refined tract-tracing studies, have provided accumulating evidence for a distinction between two types of brain network. Networks following the core synaptic hierarchy by which sensory areas project in a feedforward/feedback fashion to progressively higher-order areas, which then project to motor areas, are described as ‘canonical circuits’ [10]. Examples include sensory processing to the level of object recognition in the ventral ‘what’ pathway to inform decision making and behaviour, and processing in the dorsal ‘where’ pathway which links motion cues represented in retinotopic visual areas to the FEF to guide eye movements. Non-canonical circuits, by contrast, describe the networks formed from widely distributed regions across different levels of the sensory-fugal axis. While their nodes are reciprocally connected, they violate the local feedforward/feedback connections between adjacent areas forming the stepped processing hierarchy found in canonical circuits [10]. In other words, while the sensorimotor and association cortices are organized as a graded anatomical hierarchy in terms of cytoarchitectonic transitions between adjacent areas, connectivity based on axonal projections between areas forming circuits can be canonical (adjacently reciprocal in a stepwise hierarchy) or non-canonical (widely distributed).

The distributed axonal connections of non-canonical networks allow them to span the sensory-fugal axis of association cortices, which in turn underpins their pre-eminent role in the regulative hierarchy of cognition and behaviour [22,23]. This can be illustrated by considering two non-canonical networks. The multiple demand network (MDN) overlaps with what has previously been termed the ‘frontoparietal’ or ‘central executive’ networks, linking core regions in prefrontal, parietal, cingulate and insular cortices, in which dorsolateral PFC remains a critical hub. MDN supports executive functions of attention, working memory, response selection and inhibition, which in turn enable reasoning, decision making and hierarchically structured thought. By contrast, the default mode network (DMN) links cortical midline regions of medial PFC and posterior cingulate cortex/ precuneus, with medial temporal regions, including hippocampus and parahippocampal gyrus, and dorsal regions, including dorsomedial PFC, temporoparietal junction (TPJ) and lateral temporal cortex. DMN is involved in semantic processing, autobiographical memory, reflexive awareness, mental time travel and affectively laden representations of the self in relation to others [22–24]. These differing functional profiles are reflected in meta-analytic decoding of cognitive terms associated with each network, situating them on a sensorimotor-association cortical gradient of concrete-to-abstract cognitive domains [22–24,29,30]. Similarly, studies of semantic processing show a principal semantic gradient in which perceptual and physical categories are linked to sensorimotor areas, while more abstract concepts relating to emotions and social interactions are linked to transmodal cortices extending to DMN regions [31]. The concrete-to-abstract gradient is accompanied by a temporal gradient in information processing. Specifically, studies of brain activity during movie-watching show that the length of events represented in brain regions varies from short events in sensory areas to long events in transmodal areas. This has led to the proposal that cognition involves ‘temporal receptive windows’ by analogy with spatial receptive fields, indicating the period within which previous information can influence the interpretation of novel sensory input. Temporal receptive fields vary from milliseconds to seconds in primary sensory areas tracking dynamic changes, to seconds, minutes and longer periods in transmodal association areas as they encode more temporally extended features of the world [24,32].

The functional profiles of MDN and DMN are reflected in their respective cortical types. Both networks span eulaminate to agranular regions along the sensory-fugal axis, therefore selectively linking unimodal and transmodal association cortices to rapidly integrate and transform widely distributed information across the cortical sheet. Nevertheless, while both the MDN and DMN overlap along the sensory-fugal axis, they differ in the proportions of each cortical type that they link [22]. The DMN is dominated by cortical types that are connectionally and functionally further from sensory areas than the MDN, with a more balanced distribution of unimodal, heteromodal and agranular cortex. As such, the cortical types of the DMN are maximally ‘distant from input’, sitting at the extreme end of the macroscale gradations from sensorimotor to transmodal cortex [22,30]. By contrast, the MDN is principally formed from cortical types between the DMN and sensory areas, with a predominance of heteromodal eulaminate cortex [22]. These differences likely reflect the greater involvement of DMN in affectively laden, conceptually rich and perceptually uncoupled mental states, which influence motivation and high-level goals, involving a topographical shift of brain activity away from sensorimotor regions towards the transmodal end of the gradient [22,30]. This contrasts with the greater involvement of MDN in task organization and problem solving in practical contexts with a correspondingly greater functional and connectional proximity to sensorimotor cortices.

### (d) Non-canonical networks and distinctively human cognition

Moving up the cortical hierarchy is therefore linked to larger receptive field size, longer response latencies, multi-modal integration, conceptual abstraction and increasing temporal duration of sensory and cognitive processing [19]. The non-canonical networks supporting higher-order cognition link association cortices but not primary sensory or motor cortex, emphasizing the centrality to mammalian and primate cognition of flexible, abstract, temporally extended processing intervening between sensory input and motor output [10,11,18,22]. Human cognition is characterized by the ‘runaway evolution’ of this intermediate processing, reflected in the enormous expansion of association cortices in hominid evolution compared to other primates and mammals more broadly [10,11,33]. Mesulam emphasized how this intermediate processing creates ‘a neural buffer between stimulus and response in a way that delays closure so that novel associations and alternative responses can be considered’ [17, p. 376]. This underpins specific features of human cognition which are also implicated in response to suggestion and functional symptoms.

Human cognition is symbolic in character, involving an ability to construct abstract or ‘higher-order’ conditional associations in which sign stimuli and/or mental representations evoke meaning in terms of an implicit system of relationships between them [33,34]. Abstract symbolic processing dominates human cognition and enables the attribution of not only ‘the significance rather than the surface properties of sensory events’ [11, p. 1013] but also the meaningfulness of mental representations generated independently of sign stimuli or other immediate sensory information. This capacity for perceptually uncoupled or ‘off-line’ representation, along with conceptual abstraction and creativity, the representation of mental states of self and others and signal systems and conventions for their use in communication, are all components of symbolic cognition in humans [10,11,18,22]. In turn, this suite of abilities enables the communication and learning of the shared meanings and behaviours that we call ‘culture’, including ideas and expectations relating to normal and abnormal psychological and bodily functioning.

Suggested effects and functional symptoms are influenced by, and in some cases precisely conform to, symbol-based mental representations (where ‘symbolism’ encompasses verbal and non-verbal mental representations which form part of larger systems of meaning and interpretation). This is most obvious in the case of verbally administered suggestions, where the participant attends to the semantic content of the suggestion [9]. A constructive response style involves conscious engagement with the content of the suggestion by enlisting and attending to relevant imagery which is held in working memory to generate the suggested effect [35]. A concentrative response style also involves attention to the semantic content of the suggestion, but at a phenomenological level the suggested effect is experienced as ‘happening by itself’ without the requirement for a more conscious and agentive ‘working with’ the suggestion [35]. Patient reports of functional symptoms also tend to depict them as ‘happening by themselves’. Rather than the explicit semantic content of verbal suggestions, functional symptoms often conform to implicit ideas about illness, reflecting widespread cultural models of illness or more idiosyncratic learning based on observation and descriptions of symptoms (as in the case of a person with epilepsy who develops non-epileptic seizures influenced by descriptions of their epileptic seizures) [35,36]. Patients also sometimes describe variations in mental state which influence whether or not a symptom will occur [2]. This can be present when a symptom originates—for example, anxious attention to and appraisal of bodily sensations as portending disability following an accident—but also when symptoms become established. Indeed, this subjective recognition can provide targets for therapeutic interventions to reduce functional symptoms using methods such as therapeutic suggestion or CBT [2]. As such, there are likely to be at least partial analogues of both constructive and concentrative processes in the generation of functional symptoms, even if the latter tend to involve a greater degree of implicit learning and automatic responses to internal and external cues. For present purposes, the key point is that variations in mental state involving non-canonical (distributed) networks are implicated in the generation and modulation of suggested effects and functional symptoms.

### (e) Higher-order cognition as context and constraint for sensorimotor processing

Here we consider the proposal that suggested effects and functional symptoms produce experiential changes by heightened attention to relevant mental content. While the mechanisms are partially distinct, both have the net effect of constructing and privileging a cognitive set in the control hierarchy of cognition, which then influences signal processing across the cortical hierarchy (such cognitive sets would be termed ‘rogue representations’ in Brown's model of medically unexplained symptoms [37], and Brown & Oakley's integrative theory of suggestions in hypnosis [35]). Understanding what types of higher-order processes are potentially involved in suggested effects and functional symptoms can be approached through evolving understandings of mental states and their links to distributed networks interacting with subcortical and neuromodulatory systems [38].

Spontaneous mental states form a family of transient cognitive or emotional states which vary in content (what a given state is about) and how the subject relates to them (such as perceiving, believing, fearing, imagining or remembering) [38]. Mental states form meaningful sequences which involve the self as subject of experience and agent of action, a ‘centre of narrative gravity’ [39], while recognizing that the sense of self can alter or attenuate both as part of normative variation and psychopathology [40]. Mental states vary in the extent to which deliberate and automatic constraints influence their content and transitions. Cognitive control involves deliberate, goal-directed guidance of thoughts, perceptions and actions by executive networks (such as MDN) [38]. Automatic constraints, by contrast, operate outside cognitive control through ‘bottom-up’ processes, such as affective salience in response to emotive stimuli, or sensory salience through features such as high perceptual contrast. Automatic constraints on mental states are very diverse, originating in systems such as the ventral attention network, salience network, divisions of the DMN and limbic regions such as the amygdala (see [25] and [38] for further delineation). A further source of automatic constraints through ‘habits of attention’ involves cortico–thalamic–striatal circuits [25,41] while neuromodulatory influences on mental states occur via noradrenaline, dopaminergic and cholinergic systems [25].

The respective balance between cognitive control and automatic constraints varies with different mental states. Deliberate constraints are minimal during dreaming, and progressively increase in the transition from mind-wandering to creative thinking and then goal-directed thought [38]. Mental states also vary in their degree of abstraction (from sensory imagery to solving an anagram), affective salience, focus on social or non-social topics, and the self. Within this variation, a typical hypnotic induction procedure involves suggestions for attention and relaxation followed by the administration of specific suggestions [9,42]. In keeping with this, activity in DMN reduces while executive regions increase activity with greater depth of hypnosis (‘trance’) in which attention is focused and discursive thought and reflexive awareness attenuate. Opposite activation patterns occur during ‘mind-wandering’ when thinking is uncoupled from external stimuli [43]. The induction procedure therefore establishes a goal-directed state of deliberately heightened cognitive control and reduced influence of automatic constraints such as distraction by salient thoughts or stimuli. While the state involves cognitive control, at a phenomenological level it is experienced as a calm, undistracted state of effortless attentional immersion—‘absorption’ [43]. The use of a hypnotic induction procedure is associated with a small but significant increase in response to additional suggestions compared to the administration of suggestions without a prior induction procedure (so-called ‘imaginative suggestibility’) [44]. This is most likely owing to establishing a context and state in which heightened attention can be applied to further suggestions, along with increased expectation of response to suggestions by labelling a procedure as ‘hypnosis’ [45]. Attention to the content of suggestions is followed by congruent experiential changes accompanied by alterations in brain activity, which persist until the suggestion is reversed. Numerous neuroimaging studies have shown how brain activity varies with the content of the suggestion [46,47]. The distinction between constructive and concentrative response styles relates to variations in the degree to which a cognitive set of relevant imagery and associations has to be deliberately assembled, held in working memory, and appraised for a suggested effect to occur, as opposed to attention to the content of the suggestion eliciting pre-conscious changes at a nonetheless high level of the control hierarchy [35]. Nevertheless, both types of response style involve direct attention to the content of a suggestion which generates a relevant cognitive set in high-level control regions.

By analogy with suggestions, the hypothesis here is that the generation of functional symptoms involves attention to a relevant cognitive set. However, unlike the combination of high cognitive control and low automatic constraints that are characteristic of response to suggestions, functional symptoms involve attentional capture and cognitive appraisal which (in many cases) are motivated by the emotional salience of the relevant ideas and imagery. Cognitive control is low in the sense that it is difficult to direct attention away from a focus on emotive preoccupations (where the relevant mental representation is an object of awareness). This would be present, for example, in a patient with acute muscular spasm and bruising after falling on their back, who fearfully imagines that they have been paralysed while being taken to hospital and awaiting assessment (see also the classic clinical vignette of Reynolds [48] also summarized in Binet & Fere [49]). Despite differences in the respective contribution of deliberate and automatic constraints on cognition, in this model suggested effects and functional symptoms both involve the construction of and attention to a cognitive set capable of influencing lower levels of the cortical hierarchy. In this clinical example the combination of imagery and expectations relating to disability, combined with intense emotional arousal, may rapidly consolidate novel sensorimotor representations which influence subsequent functioning even if the patient wants to accept medical reassurance that recovery will be complete. This is in contrast to suggested effects that are readily reversible because they combine high cognitive control, low emotional salience and an underlying epistemic attitude that the content of the suggestion is imaginary rather than real.

There is, however, unlikely to be a single neural signature for heightened attention to a cognitive set generating functional symptoms for several reasons. As noted above, there are different types of automatic constraints on cognition. Some automatic processes may motivate attention to relevant imagery and attributions without intense, acute emotional arousal (for example, habitual self-monitoring in the presence of established expectations of symptoms) [37]. There may be differences in the extent to which the content of a cognitive set is constructed as a novel set of representations held in working memory, compared with previously learned symptom schemata established in implicit memory which can influence lower-order processing. In this respect, illness models for functional symptoms may be strongly influenced by personal experience. This may occur, for example, with exposure to illness behaviour and descriptions in the family setting from an early age (see [37] for a detailed mapping of different sources of ‘rogue representations’). The psychodynamic concept of ‘cathexis’ refers to how mental representations are invested with emotional significance, and can be applied to how certain illness representations are developmentally acquired and gain special cognitive influence. The cathexis of illness representations and the differing contributions of cognitive control and automatic constraints in suggested effects and functional symptoms also help explain why there is only a moderate correlation between hypnotic suggestibility and functional symptoms [50]. Highly hypnotically responsive individuals have a talent for engaging with a wide range of suggestions and responding to them through high levels of cognitive control. Individuals who are not highly hypnotically responsive may nevertheless display specific functional symptoms based on illness representations invested with emotional significance [37]. Such representations are implicitly learned, associatively linked to triggering cues and acquire a low threshold for activation under relevant stimulus conditions.

A key challenge is to understand how attentionally salient mental representations can influence experience through effects on brain function [51]. What Mesulam described as ‘our highly edited subjective version of the world’ [11, p. 1013] arises at a late stage of cognitive processing. Anything that alters the pre-conscious ‘editing’ of experience can therefore alter what we experience. Here, we consider different models of how specific representations privileged by heightened attention can influence signal processing in the cortical hierarchy to generate suggested effects and functional symptoms.

## 3. Hierarchical predictive coding

Hierarchical predictive coding (HPC) has emerged as an influential account of how high-level representations constrain processing across the cortical hierarchy since its initial exposition by Karl Friston and subsequent development [52,53]. Among many other conditions, HPC has been applied to functional neurological symptoms [54] and suggestions in hypnosis [55,56]. In developing HPC, Friston applied Bayes’ theory of conditional probability to Mesulam's account of the nervous system as a synaptic hierarchy linking sensory surfaces to higher-level processing over successive synaptic steps [55]. In this model, backward connections provide contextual guidance based on prior knowledge from each level to the preceding level through prediction of the lower level's inputs. Each prior is based on ‘empirical Bayes’, where expectations acquired from sensory data through Hebbian learning represent a ‘regularised solution’ to probable causes of sensory input under similar contextual conditions. The prediction based on a prior at each level of the hierarchy is represented in backward and lateral projections to form a generative or ‘forward’ model for the preceding level. When the prediction is incompatible with input from the lower level, an error message (prediction error) is generated and passed back via forward connections to the level above. The prediction error elicits an adjustment of the prediction from the level above until reconciliation between the prediction and the input is achieved. The adjustment involves interactions with adjacent neuronal populations encoding priors at the same level via lateral connections until a more accurate prediction is selected which matches the sensory data. Selection is, however, critically biased by the ‘precision’ or inferential weight of a given prior. A precise prior is more likely to be selected and then influence what error messages from the lower level are ‘listened to’ [53]. The ‘posterior probability’ resulting from the consensus between prior and input corresponds to perception. Any process that amplifies the precision of priors or prediction errors can therefore profoundly affect what is perceived. Precision is enhanced by attention, which as noted above can increase with cognitive control or a range of automatic processes, which increase synaptic gain through neuromodulation [53,54]. The precision of priors also of course reflects their consolidation in neuronal populations through previous Hebbian learning.

Prediction error reconciliation has been described as a form of ‘belief updating’. As Friston put it in a recent account of HPC [53, p. 257], ‘beliefs here are Bayesian beliefs, namely, subpersonal or propositional representations that possess the attribute of uncertainty, and are conditional in a well-specified sense. Under this reading of beliefs, sentient behaviour—and beliefs about that behaviour— can then be cast as inference (e.g. perceptual inference, planning as inference, attribution of agency, emotional recognition, etc.). Reducing everything to inference then provides the mechanics to understand neuronal dynamics and development in terms of belief updating and the requisite message passing in the brain [53]. The informational content of a ‘Bayesian belief’ or prior therefore varies with the levels of the cortical hierarchy in which it is implemented. Attentionally salient high-level representations, such as a verbal suggestion in hypnosis or an anxious conviction that an accident has caused paralysis, can both be thought of as types of belief involving mental representations that are or could be conveyed as verbal propositions. They can also act as ‘Bayesian beliefs’ or priors at a high level of the cortical hierarchy as a basis for influencing lower levels through backward projections. However, the informational content of ‘beliefs’ in the Bayesian sense of HPC radically changes in successive steps down the cortical hierarchy, and so are not beliefs in the usual propositional or psychological sense of that term. Friston referred to these subpersonal informational states as ‘representations’ rather than ‘beliefs’ in his initial exposition of the theory [52], commenting:

Here, a representation is taken to be a neuronal response that represents some ‘cause’ in the sensorium. Causes are simply the states of processes generating sensory data. It is not easy to ascribe meaning to these states without appealing to the way that we categorize things, either perceptually or conceptually. Causes may be categorical in nature, such as the identity of a face or the semantic category to which an object belongs. Others may be parametric, such as the position of an object. Even though causes may be difficult to describe they are easy to define operationally [i.e. mathematically]. Causes are quantities or states that are necessary to specify the products of a process generating sensory information [52, pp. 819–820].

Consequently, when HPC is defined as belief updating through prediction error reconciliation, successive levels in the cortical hierarchy involved in sensation are considered to integrate bottom-up prediction errors related to incoming sensory data and top-down priors (Bayesian beliefs) about the causes of sensory data in an optimal way. The interaction between the likelihood of sensory data and the prior beliefs over those data at relevant levels results in a posterior distribution corresponding to the posterior belief or percept. In this model superficial pyramidal cell populations encode prediction error at the lower level, and deep pyramidal cells encode posterior expectations at the higher level [53,54]. The posterior expectation corresponding to a perception represents the point of reconciliation between top-down priors and bottom-up sensory data when prediction errors are ‘resolved’.

HPC therefore raises the question of the level of the cortical hierarchy at which perception is predominantly decided. Processing to the level of object recognition occurs in well-defined steps, which lend themselves to rapid perceptual resolution in local loops. Basic processing, such as recognizing a simple line orientation, occurs between early visual areas (V1 and V2), while more complex visual feature processing culminating in face and object recognition is distributed across the ventral visual stream extending to occipital extrastriate areas and temporal regions (such as inferior temporal, fusiform and parahippocampal regions). Further projection of visual processing streams to diverse transmodal regions and networks supports a range of cognitive functions relating to social and emotional cognition, memory, language processing and spatially directed attention [57,58].

Perception is usually highly automatic and relatively insensitive to higher-order influence. The resolution of perception at early levels in the processing hierarchy underpins sensory illusions, which present as a perceptual ‘given’ even with explicit knowledge of the underlying stimulus characteristics. Nevertheless, in the HPC framework other levels of the cortical hierarchy and greater reliance on priors relative to prediction error influence perception under certain conditions. This occurs, for example, with sensory degradation or ambiguity, such as interpreting speech in a noisy environment by attending to facial expression and mouth movements [58]. Other examples occur in functional neurological symptoms and suggested effects in hypnosis.

### (a) A Bayesian approach to functional neurological symptoms

In ‘A Bayesian approach to Hysteria’ [54], Edwards and colleagues applied HPC to the explanation of functional motor and sensory symptoms (FMSS). They proposed that ‘the fundamental pathology in FMSS is the presence of overly precise priors at intermediate levels of the hierarchy, which may lead to the overweighting of bottom-up inputs that accord with those priors’ [54, p. 3508]. For example, an abnormal prior belief related to sensation in a relevant domain (such as the insular cortex for pain and the secondary sensory cortex (SII) for non-painful sensation) is afforded too much precision via maladaptive attentional gain from higher hierarchical levels. The increase in precision (synaptic gain) shifts the posterior probability towards the prior expectation, which ‘overwhelms’ the influence of bottom-up prediction errors. The resulting perception conforms to the prior beliefs encoded at the intermediate level, which is ‘impervious to bottom-up prediction errors’. In turn, a precise prediction error is propagated to higher levels, which biases the selection of priors to achieve reconciliation at successive levels. At a phenomenological level, this corresponds to the functional symptom in the relevant domain, such as increased pain or decreased sensation. Symptoms are typically accompanied by high-level beliefs in the veridicality of the symptom and causal attributions that attempt to make sense of it.

In this account, a functional symptom such as anaesthesia requires the pre-existence of an abnormal prior belief at a relevant level, such as SII. The high precision of the abnormal prior can be based on previous learning, or acutely enhanced by attention to that area. A different prior represented in SII would lead to a different perception. The authors comment, ‘FMSS can be seen as an extreme instance of a common phenomenon in predictive coding networks—the overweighting of prior beliefs over sensory data—which underlies normal experiences from optical illusions to placebo effects’ [54, p. 3508].

This framework is applied to functional motor symptoms, such as involuntary movements or paralysis. HPC proposes that normal movements involve a process of ‘active inference’. Predictions about the sensory consequences of intended movement are hypothesised to arise at a high hierarchical level, such as the pre-supplementary motor area (pre-SMA), which is active during conscious intention and planning to move [54]. Predictions are transmitted down the motor hierarchy, producing a proprioceptive prediction error (relating to the position and motion of the limbs) that is resolved by movement. In other words, movements are implemented in motor hierarchies to remove proprioceptive prediction errors, by analogy with how specific perceptions arise to remove prediction errors in sensory hierarchies. In the case of functional motor symptoms, the hypothesis is that an abnormal prior expectation related to movement implementation is formed within an intermediate motor area. The SMA is a candidate region, given that it is active during motor planning and execution but not with conscious intention to move *per se* [54]. The abnormal prior is assigned abnormal precision (synaptic gain) by attentional processes. In turn, this causes motor predictions activated in intermediate levels to elicit movement. These unconsciously generated movements have different prediction errors from those that would have been generated at higher conscious levels (associated with engagement of pre-SMA). Salient prediction errors relating to unpredicted movement are returned to high-level motor control regions via sensory-fugal connections, which are experienced and appraised as involuntary. In this model functional paralysis also results from an abnormal prior at intermediate levels of the motor hierarchy making predictions of movement. In this case the prior predicts inability to move, which overrides the propagation of conscious intentions to move originating at higher levels. The resulting prediction errors are transmitted to higher-level motor regions and consciously experienced and appraised as limb paralysis. Distinct abnormal priors relating to movement can explain why the same patient can have both functional limb movements (seizures and tremor) and paralysis.

This exposition of HPC and its application to functional symptoms has emphasized exteroceptive processing streams (e.g. visual, touch and pain) and proprioception (position and motion of limbs in relation to motor function). However, HPC is proposed to act across all sensory processing streams, including interoceptive processing relating to sensation of internal bodily states, such as hunger, thirst, fullness and temperature [53,54]. Indeed, modulation of interoceptive processing in which the anterior insula and anterior cingulate cortex are key nodes is central to Brown and colleagues’ predictive coding model of functional somatic (‘medically unexplained’) symptoms [59]. This approach assumes that functional somatic symptom reports are uncoupled from sensory input and arise when following predictive cues ‘excessive precision is afforded to priors predicting the presence of symptoms/disease, rendering those priors the optimal model regardless of whether they are a good match for sensory input’ [59, p. 194].

### (b) Bayesian approaches to suggested effects

The mechanisms of suggestion have also been interpreted in terms of HPC. Jamieson [55] proposed that suggestions work by attenuating sensitivity to interoceptive prediction errors through reduced activity in ventral anterior insula. This reduces input to the dorsal anterior insula, which would otherwise drive attention to salient stimuli and help motivate goal-directed behaviour. This, in turn, lessens the ‘pressure’ to update current predictive representations introduced by suggestions, allowing ‘extended periods of stability to emerge in the activity of the multilevel networks comprising the generative model’ [55, p. 328]. In this view, suggested effects work not only by the introduction of suggested priors, but also by the attenuation of interoceptive responses that would otherwise contradict them—the basis of the distinct ‘reality orientation’ established by suggestion.

An alternative application of HPC to mechanisms of suggestion has focused on motor suggestions [56]. This theory proposes that motor suggestions increase the precision of both proprioceptive predictions and actual proprioceptive evidence by attentional modulation. The resulting mismatch between predictions and sensory evidence calls for an explanation, which is provided by the attribution of external agency within the suggestion.

This brief exposition of differing applications of HPC illustrates proposals for how representations privileged by heightened attention can influence signal processing in the cortical hierarchy to generate suggested effects and functional symptoms. There is scope for more detailed consideration of each set of proposals [60]. However, in general all of these accounts seek to explain specific functional or suggested effects through belief updating by prediction error reconciliation implemented across the cortical hierarchy, as modelled with Bayes’ theory of conditional probabilities. All propose that specific priors are ‘overweighted’ (increase precision): for functional symptoms at intermediate (non-conscious) levels of the cortical hierarchy [54], and for suggested effects at higher levels representing the ideational content of suggestions [55,56]. This works to override processing of sensory prediction errors (sensory functional symptoms); alter proprioceptive prediction errors eliciting attributions of loss of self-agency (functional motor symptoms) [54]; attenuate processing of interoceptive prediction errors allowing suggested priors of all types to propagate without correction [55,56]; or increase processing of proprioceptive prediction errors for suggested motor effects to elicit altered attributions of agency in keeping with suggested priors [56].

Differences in these models underline how HPC as a general framework is applied to hypotheses about specific brain regions and networks involved in altered perceptions and self-experience. Key questions concern which cortical networks, axes, levels and patterns of top-down and bottom-up influences are involved. The models also raise the question of whether all cognitive processes, and all relationships across regions and networks within the cortical hierarchy, lend themselves to explanation in terms of HPC. To consider these questions, we will consider some alternative models of neurocognitive function and their implications for understanding suggested effects and functional symptoms. We will then consider these models in light of HPC, and their collective implications for understanding suggestion, functional symptoms and their potential interactions with VR.

## 4. Top-down cortical feedback pathways and signal routing

Mesulam used the term ‘transcendent encoding’ to refer to perceptually uncoupled, ‘off-line’ representations of events that are not confined to the sensory features of an experience [17]. Mesulam regarded this mode of cognitive activity as distinct from the predictable sensorimotor sequences that lend themselves to predictive coding. He also identified ‘contingent routing’ as the ability to link one stimulus to multiple possible responses, requiring a conditional suppression of prepotent responses to allow contextual significance and internal priorities to be worked out. Zagha [19] has extended this insight in his development of a framework for understanding how prefrontal and other neocortical regions influence sensorimotor processing. In this model, cortical feedback to lower levels of the cortical hierarchy routes sensory and motor signals according to goal-direction and context represented at higher cortical levels. This occurs via backward pyramidal neuron projections forming excitatory synapses onto lower-level pyramidal neurons or interneurons, which variously disinhibit or inhibit pyramidal neurons depending on their properties. The net result is context-dependent patterns of excitation and inhibition in lower target regions, resulting in the activation of relevant neural ensembles and suppression of others. This modulates activity within regions and the coupling strength between regions, so that the same signal may be routed to diverse targets, or not propagated should it be inhibited. Top-down pyramidal projections are considered the primary regulators of forward signal propagation because of the speed and anatomical precision of their targeted effects, even if ascending neuromodulatory pathways (such as those releasing norepinephrine, dopamine, serotonin or acetylcholine) additionally influence forward transmission.

Cortical feedback across multiple hierarchical levels distributes contextual signals throughout the cortex, so conveying the conditions for relevant routing of goal-directed behaviour before stimuli arrive. This enables rapid, diverse patterns of signal propagation to create a wide range of novel sensory–motor associations in keeping with internal priorities and contextual demands. On this basis Zagha [19] described cortical feedback pathways as ‘proactive regulators of feedforward signal propagation’. The determination of context and priorities emerges from the broad range of mental states supported by non-canonical distributed networks and their interactions, from dreaming and mind-wandering to creativity and problem solving. The states vary in their topographical distribution along sensorimotor-transmodal gradients, differing in the involvement of emotion, perceptual uncoupling, abstraction and social or non-social focus, and the relative influences of cognitive control and automatic constraints on cognition [38].

Zagha [19] also proposed an anatomical framework for how different regions and levels of the cortical hierarchy are involved in different aspects of sensorimotor function. It provides an alternative to HPC for analysing how specific representations privileged by heightened attention can influence signal processing in the cortical hierarchy to generate suggested effects and functional symptoms. Here we explore the utility of this anatomical framework by using it to interpret findings from neuroimaging studies of suggested effects and functional symptoms.

### (a) Analysis of functional symptoms and suggested effects in terms of cortical source, target and function

#### (i) Prefrontal cortices to motor cortices (motor preparation and impulse control)

Zagha summarizes evidence for a motor control hierarchy. Feedback projections from PFC convey aspects of task rules and action selection to the premotor cortex, which prepares a detailed motor plan. Once trigger conditions are met, the motor cortex initiates the prepared motor plan. Neuroimaging studies suggest that these relationships are altered in functional motor symptoms and suggested effects. In the case of functional limb paralysis and its modelling with suggestion, some studies suggest that paralysis results from prefrontal executive inhibition intervening between motor intention and planning and movement execution [61–64].

By contrast, Cojan *et al.* [65] did not find evidence of increased motor inhibition from prefrontal regions during functional paralysis or its experimental modelling with suggestion. Instead, they reported increased functional connectivity between the motor cortex and precuneus, proposing that in both cases motor inhibition (paralysis) may be mediated through mental imagery and self-reflective processing rather than executive inhibition. Also, functional paralysis but not suggested paralysis was associated with modulation of ventromedial PFC [65,66]. They interpreted this as involvement of affectively laden self-representations and memories in modulating motor function during functional but not during suggested paralysis. In terms of Zagha's anatomical framework, all studies involved feedback modulation of motor cortices by prefrontal and other neocortical regions even if the studies differ with respect to the role of inhibition and motivation in the experience of paralysis.

Involuntary movements occurring as functional symptoms and suggested effects also modify activity within and between components of the motor control hierarchy. Walsh *et al.* [67] used suggestion in 18 highly hypnotically responsive individuals to produce thought insertion and alien control of movement in an automatic writing paradigm. Both thought insertion and alien control of movement involved a reduction in activity of left SMA and were associated with altered functional connectivity between SMA and brain regions involved in language processing and movement respectively. Collectively, these results were interpreted as indicating that SMA plays a central role in alien control phenomena as a high-level executive system involved in the sense of control and ownership of thoughts and movements [67]. These findings illustrate alterations in the modulatory activity of a premotor region (e.g. SMA) linked to a prefrontal region (e.g. pre-SMA) in relation to motor cortices in the case of alien control of movement. However, thought insertion and alien control of movement were clearly differentiated in the relations between motor cortices and sensory cortices.

#### (ii) Motor cortices to sensory cortices (corollary discharge)

The forward model of motor control is an early version of a predictive coding model, which proposes that voluntary movement initiation is accompanied by a corollary discharge to sensory cortices (an ‘efferent copy’). The predicted sensory signals are subtracted from the sensory input resulting from movement, cancelling out the reafferent (self-generated) component. This results in attenuation of sensory awareness of self-generated movement, preventing distraction by redundant sensory information and helping to distinguish self-initiated bodily movement from that due to involuntary causes. In the automatic writing study of Walsh *et al.* [67], alien control of writing movement was associated with increased activity of the left cerebellum during movement preparation and the left angular gyrus during movement implementation. These findings extended those of an earlier PET study of suggested involuntary movement [68] by evidencing a failure to attenuate activity in sensory processing regions during both the preparation and implementation phases of movement. Together, these findings were consistent with a forward model account in which suggested involuntary movement is associated with a failure to attenuate sensory processing of what in reality are self-generated movements. Zagha's analysis of reciprocal relations between motor and sensory cortices during the experience of movement can also be interpreted in terms of HPC. In the HPC framework, the failure to suppress sensory processing regions during suggested involuntary movement can be viewed as unresolved prediction errors which reflect, and may contribute to, the feeling of involuntariness during what in reality are self-generated movements. This shows how suggestions of movement control by an external agent have effects that cascade through the motor control hierarchy. Nevertheless, the thought insertion condition in the automatic writing paradigm of Walsh *et al.* [67] forms an important contrast to the alien control of movement condition in terms of brain mechanisms involved in the sense of control and ownership - both in terms of Zagha's use of a forward model to understand relations between motor and sensory cortices, and by extension to HPC. In the automatic writing paradigm, thought insertion—the attribution and experience that thinking of a sentence stem ending was inserted into the mind by an external agent—was associated with reduced activation of networks supporting language, movement and self-related processing. Thought insertion was not, however, associated with increased activity in brain regions involved in sensory processing analogous to the failure to attenuate cerebellar and angular gyrus activation in the case of alien control of movement. This type of linguistic thought, unlike movement, does not have well-defined sensorimotor characteristics. In the absence of sensorimotor characteristics, forward modelling of thought does not appear to occur. This suggests that the sense of control and ownership of thought does not involve modulation of feedback from sensory processing regions as occurs in movement. In terms of HPC, the absence of well-defined sensorimotor characteristics when thinking of a sentence ending raises the question of what could generate prediction errors at higher levels of the cortical hierarchy, assuming—as HPC appears to—that prediction error reconciliation is an obligatory feature of cortical processing. The findings for both conditions suggest that the sense of control and ownership critically involves modulation of a high-level transmodal hub (e.g. SMA), with the absence of alterations in sensory processing regions in thought insertion implying that such changes are not necessary for the sense of control and ownership *per se*, although they may contribute to an altered sensation of movement specifically.

#### (iii) Prefrontal cortices to higher-order sensory cortices (feature-based attention)

Zagha observes that top-down attention is directed by prior knowledge and goal direction and implemented by feedback pathways. He proposes a framework in which attentional signals originate in PFC and modulate the coupling strength of task-specific sensory streams via feedback pathways. These changes in coupling determine whether a stimulus is attended (high coupling, propagated to higher-order sensory cortices) or ignored (low coupling, prevented from propagating to higher-order sensory cortices).

In this context functional and suggested hyperalgesia, and analgesia, represent an accentuation of normal processes in which forward transmission of pain sensation is prioritized or inhibited. Neuroimaging findings from suggested and functional (nociplastic) fibromyalgia (FM) pain show that pain experience can be accentuated by top-down modulation. Also, the findings of Derbyshire *et al.* [69] and other studies of functional (nociplastic) pain [70] suggest that cortical levels representing pain can be modulated to generate pain-related experience irrespective of peripheral sensation (noting that this was also proposed by Edwards *et al.* [54] in their HPC model). The studies of suggested pain [69] and FM pain [70] show a partial overlap in activity in pain matrix components of the insula and ACC during pain processing. Differences may partly relate to differences in the respective contributions of cognitive control and automatic constraints on privileging high-level representations that modulate brain activity, with greater involvement of brain regions involved in emotional arousal, motivation and salience in functional symptoms. Also, differences in brain activity may reflect neuroadaptational changes owing to the chronicity of functional symptoms, compared with the temporary and reversible nature of suggested symptoms.

#### (iv) Higher sensory cortices to lower sensory cortices (predictive coding and sensory perception)

The final category of cortical feedback proposed in Zagha's anatomical framework is from higher-order sensory to lower-order sensory cortices. Perception is analysed in terms of Mumford's (1992) non-Bayesian account of predictive coding in which predictions about sensory inputs are derived from prior knowledge about the sensory content of our surroundings [71]. This account—similar to the application of HPC to perception—posits that sensory predictions from higher-order sensory cortices cancel out predicted patterns of sensory input from lower-order sensory cortices. The remaining prediction errors elicit alterations in predictions from higher levels, until expectations match sensory evidence to result in perception [71]. In this framework sensory inputs from VR, which simulate veridical sensory input, enlist brain systems involved in perception to generate VR experiences.

In summary, Zagha's model of top-down cortical feedback and signal routing, and anatomical framework for analysing experiences of perception and movement in terms of cortical source, target and function, can be applied to the interpretation of brain mechanisms involved in suggested effects, functional symptoms and VR experiences. Its use of a model of the cortical hierarchy, top-down modulation and forward signal transmission and partial use of predictive coding, raises the question of how it relates to applications of HPC to suggested effects, functional symptoms and perception (in the case of VR). We have considered links between Zagha's approach and HPC in relation to experimental studies of suggestions for alien control of movement and thought insertion. We will further consider the links between Zagha's approach and HPC after considering another model of cortical modulation with implications for understanding similarities and differences between suggested effects and functional symptoms, particularly in terms of experience-dependent changes in brain networks—attractor dynamics.

## 5. Attractor dynamics and their application to suggested effects and functional symptoms

An attractor network is ‘a neural network that can occupy numerous stable states that are learned from experience, via adjustments to synaptic weights. It can revisit these states if presented with inputs that resemble previous patterns of synaptic weights, or through spontaneous fluctuations in neural activity; either way, the activity of all nodes is ‘attracted’ to a quasi-stable state because the network energy is lower at these states, and network firing patterns evolve to minimise energy’ [72, p. 9472].

There are different models of attractor networks used to explain diverse aspects of brain function, such as eye movements or head direction [73]. Here we focus on attractor models which are most relevant to understanding mechanisms of suggested effects and functional symptoms. To date these models have been used to account for perception and action control across the cortical hierarchy, as well as for neuropsychiatric conditions such as schizophrenia and obsessive-compulsive disorder (OCD) [25].

Attractor states in these ‘Hopfield-like’ networks are formed from pyramidal neurons in the cerebral cortex, which have a relatively high density of excitatory connections to each other within a local area of 1–3 mm [25,74]. Attractor networks involve a subset of axons looping back to synapse with their originating neurons. These recurrent collateral axons form excitatory synaptic connections which are associatively modifiable. This ‘autoassociation’ type of attractor network is capable of maintaining persistent activity through positive feedback. The information state of the attractor is formed by the set of neurons in the local cortical network that were coactive when the memory was formed, resulting in strengthened excitatory connections between that set of neurons. This forms the basis for reactivation of the network—and hence recall—under similar external input conditions. The informational content of the attractor could correspond to memories, perceptual representations or thoughts depending on the cortical system that it is embedded within [25].

GABA inhibitory interneurons are also involved in the selection of the most stable attractor state, ‘the basin of attraction’, from among alternative attractors that might be partially activated by retrieval cues (inputs from other neurons resembling an earlier pattern). Input to a cortical area elicits activation in pyramidal cells belonging to different attractors that potentially or partially fit with the input conditions. Higher firing rates in a ‘good fit’ attractor are also associated with stronger negative feedback from GABA inhibitory interneurons onto other excitatory pyramidal cells in the network. The combination of positive feedback via recurrent collateral connections and competitive inhibition of alternative attractors implemented through the inhibitory interneurons results in the selection of a winning network implementing a single memory state [25].

Rolls proposes that attractor networks are fundamental design features of the neocortex and hippocampal cortex but not of other regions, such as the cerebellar cortex or basal ganglia. Indeed, he suggests that ‘the presence of recurrent collateral excitatory connections between pyramidal cells is perhaps the most important characteristic of the cerebral neocortex, for they allow the formation of short term memory, and hence attention which requires a short term memory to hold the object of attention in mind, planning (which requires several steps of a plan to be held in mind), and language (which probably requires several parts of each sentence to be held online during processing)’ [75, p. 31].

Attractor dynamics vary at different cortical processing levels, reflecting the previously noted temporal gradient of information processing across the cortical hierarchy. Rolls [25] observes, for example, that early layers of the cortical hierarchy do not implement long-lasting attractor states. The increasing duration of attractor states across the cortical hierarchy is not only relevant to understanding function at a given cortical level, but also provides a basis for more enduring attractor states at higher levels to influence processing in lower levels via backprojections. Nevertheless—and in contrast to HPC—the approach emphasizes discrete processing within successive levels of the cortical hierarchy, strong forward transmission of processed information and generally weak feedback influences between levels. For example, when analysing connections between adjacent cortical areas in a sensory hierarchy as forward versus backwards, Rolls notes the separate superficial and deep laminar origins of the respective pyramidal cells. He suggests that the termination of backward axonal projections on the apical dendrites in layer-I, far from the cell bodies, indicates that the effects of backprojections are likely to be weaker than the forward connections. In his view it is in fact ‘crucial for attention and memory recall that the top-down or backprojections are weaker than the forward connections, so that attentional biasing and memory recall does not dominate over bottom-up forward inputs’ [76, 1221]. Rolls further suggests that at higher associative levels of the cortical hierarchy there are limits of top-down influences for functional reasons—for example, that short-term memory processing in prefrontal cortical areas does not dominate perceptual processing in the temporal and parietal areas that use the PFC for short-term memory. The point here is partly that adaptive cognition requires accurate guidance by sensory experience and therefore must be protected from excessive influence by internal representations. However, Rolls also emphasizes physiological constraints that limit the extent of detailed influence of higher cortical levels on lower ones. Specifically, lower levels do not represent the same information as higher levels (e.g. there are face-selective neurons in the inferior temporal cortex but not in V1). This limits the ability of higher levels to make specific predictions about the information content of lower levels. In turn, this raises the question of how detailed predictions from higher to lower levels required for prediction error reconciliation might occur, as opposed to a higher level settling into an attractor state in response to relevant afferent input. As Rolls put it,

The corticocortical back projections are not sufficiently strong to make neurons at earlier stages in the hierarchy have the same responses as at higher stages. Face selective neurons with receptive fields that can be up to 70° are not found in the primary visual cortex, where the neurons respond to features such as bars and edges and have receptive fields in the order of 1°. Instead, the cortical back projections are used for gentle influences on earlier cortical stages, such as top down attention, and memory recall if there is not a strong bottom up input. Each cortical hierarchy thus operates computationally with a feedforward style of computation, in which new representations are built at each succeeding stage, in a divide and conquer approach to computation [25, p. 70].

This draws attention to a fundamental difference between HPC and the attractor dynamic model of the cortical hierarchy proposed by Rolls and others who emphasize the dominance of feedforward connections in cortical function. As Markov & Kennedy [77, 190] put it, ‘In a Bayesian framework of hierarchical cortical computation, rather than viewing feedback as biasing competition as in the feedforward model, feedback is conceived to play the altogether more ambitious role of biasing inference.’

Rolls proposes ways in which attractor dynamics at higher levels influence processing at early levels by linking attractor networks. Short-term memories implemented in attractor networks form the basis of top-down attention by maintaining relevant information (such as spatial location or perceptual expectation) in short-term memory. These active representations influence lower-level processing through backprojections which bias competition between neuronal populations receiving bottom-up stimulation. For example, short-term memories in the PFC can bias competition in perceptual areas, such as the inferior temporal visual cortex and parietal cortex, to implement object and spatial attention. In the case of perception, sensory information is summated and integrated over successive layers of processing, and back projections enhance feature detection at lower levels to facilitate object recognition. Perception of a specific object occurs by the formation of basins of attraction that gravitate towards increasingly stable multi-modal attractors representing a best fit with sensory input based on prior learning [78]. The winning attractor network (percept) is selected through a combination of activation by afferent inputs and competitive inhibition of alternative networks over successive levels. The winning attractor is also maintained by enhanced feature detection at lower levels via back projections.

In contrast to HPC's notion of prediction error reconciliation and Zagha's model of feedback modulation, the attractor dynamics notion of mild modulatory influences from higher to lower levels in the cortical hierarchy does not seem sufficient to generate radical alterations in experience such as sensory loss, hallucinations or involuntary movements due to suggestions or as functional symptoms. However, applications of attractor dynamics to perceptual illusions, schizophrenia and OCD provide analogues for how attractor dynamics may be extended to suggested effects and functional symptoms.

The McGurk illusion, in which the auditory perception of consonants is influenced by viewing incongruent lip movements, has been modelled with a trimodular attractor architecture [25]. One module represents auditory input, another visual input, and both converge on a higher transmodal module that represents the syllable formed by combining the evidence provided by both inputs. Backprojections from the convergent module to the input modules influence their activity. The inputs and backprojections can be adjusted so that the transmodal module represents a mixture of the two inputs, or so that one or the other input wins (i.e. either the actual consonant is heard or the incongruous consonant from visual input is heard). This illustrates how visual inputs and backprojections from a transmodal convergence zone can affect what is heard via a predominantly feedforward architecture with modulatory backprojections. This model could be applied to other illusions involving more than one sensory modality, such as the rubber hand illusion in which the spatial representation of touch sensation is displaced to accord with (illusory) visual feedback [79]. This would be a case of visual information biasing the selection of an attractor representing the body in space, which in turn shifts the perceived location of touch sensation. This shows how the selection of an attractor can bias the processing of sensory information. However, in the case of the McGurk effect or rubber hand illusion the transmodal attractor is selected by visual information which is strongly weighted compared to other sensory modalities. In the case of suggested effects and functional symptoms the selection of attractors at ‘intermediate’ levels of the association cortical hierarchy is affected by top-down connections from higher cortical regions forming part of non-canonical networks rather than being driven by bottom-up inputs. Nevertheless, attractor selection supporting illusory perception can be simultaneously influenced by bottom-up and top-down effects. For example, Walsh *et al.* [79] found that hypnotic susceptibility increased responsiveness to the rubber hand illusion. Applying this to treatments combining VR with suggestion, this suggests the potential for attractor selection to be influenced both by illusory (virtual) sensory information and higher-level responsiveness to suggestions acting via top-down modulation of intermediate levels [5].

The role of top-down modulation in selecting attractors influencing perception is illustrated by a study of schizophrenia employing effective connectivity (EC) [76], which by analogy can be applied to understanding suggested effects and functional symptoms. EC was measured in resting-state fMRI acquisitions in 180 participants with schizophrenia and 208 controls. The main findings were that in cases where EC differed between the groups, on average the forward (stronger) EC in schizophrenia compared to controls was smaller, whereas the backward connectivities tended to be larger. In particular, higher EC in schizophrenia was found from the precuneus and posterior cingulate cortex (PCC) to areas such as the parahippocampal, hippocampal, temporal, fusiform and occipital cortices. These backward ECs were positively correlated with the positive symptoms of schizophrenia. Rolls *et al.* proposed that the reduced forward inputs to the posterior default mode regions of the precuneus and PCC, and increased backward projections from them to association cortices involved in perception and affectively laden processing about the self and others, ‘lead to internally generated scenarios about the self becoming detached from and dominating the forward inputs normally brought to these regions from the real world’ [76, p. 1229]. They continue, ‘Put in another way, if top-down signals are increased relative to bottom-up signals this would increase the importance of priors, that is, beliefs, at the cost of sensory signals, representing a possible mechanism for the emergence of hallucinations and delusions’ [76, p. 1230].

These findings can be placed in the context of Rolls’ modelling of attractor dynamics in schizophrenia. In the case of positive symptoms he proposes that reduced cortical inhibition, caused by a reduction of GABA neurotransmission, can lead to instability of the spontaneous firing states of cortical networks. This in turn leads to noise-induced jumps to a high firing rate attractor state in the temporal lobe even in the absence of external inputs, contributing to the positive symptoms of schizophrenia such as hallucinations and delusions [80]. In this model instability of the resultant attractor states is compounded by reduced NMDA receptor function [80]. In other words, while effective connectivity may be tilted towards a predominance of top-down signals over bottom-up signals, the representational content of symptoms may be additionally changeable owing to reduced stability of specific cortical attractors occupying ‘shallow’ basins of attraction [80]. (Conversely, fixed delusional systems or persistent auditory verbal hallucinations attributed to known persecuting agents would likely reflect overstable attractors biasing perception and its interpretation.)

Excessive stability is posited in attractor dynamic modelling of OCD [25]. The key notion is that cortical and subcortical systems involved in a particular type of stimulus appraisal and response—such as ‘dirt/cleaning’—become functionally linked to form an attractor state. In OCD, the networks become ‘too stable’: ‘once in an attractor state, the networks tend to remain there too long’ [25, p. 317]. Computational modelling shows that this could occur by increased transmission in the glutamatergic system, an excitatory system which boosts activity in the neurons it modulates. The net effect is to increase the synaptic weights (strength) between the associatively modified synapses of the network linking thought, feeling and behavioural response. The increased stability of networks and higher neuronal firing rates produce increased activity in the component brain regions, with different types of thoughts, feelings (obsessions) and behavioural responses (compulsions) depending on the regions involved.

Against this background, the premise that radical alterations of perception and appraisal involve an increase in top-down signals relative to bottom-up signals across the cortical hierarchy can be applied to attractor dynamic models of suggested effects and functional symptoms. On this basis we predict that suggestions can be viewed as requiring high levels of cognitive control, in which a representation is constructed from existing concepts (high-level attractors) and held in working memory to influence congruent lower-level systems via backprojections. Attractor states in intermediate-level systems are sufficiently activated to either reappraise bottom-up inputs or override them completely so that forward signals to other cortical systems are presented ‘as if’ sensory inputs are present or absent depending on the experiential change in question (as for example in the case of suggested or functional pain or analgesia).

Relevant questions concern the basis for the high levels of cognitive control in hypnotically responsive individuals, in whom high-level attractor states are constructed which are both highly stable (undistractable) but readily reversible, exerting an especially strong top-down influence. Individual differences in neuromodulatory systems, genes and executive function have all been invoked [81].

From this perspective functional symptoms also depend on the selection of attractor states in intermediate layers of the cortical hierarchy. As noted above, however, the representations (attractors) influencing functional symptoms are more likely to be constructed from automatic and emotionally salient cognitive processes (emotive mental imagery, beliefs and expectations) than the more cognitively controlled processes constructing responses to suggestions in hypnosis. Prior implicit learning may also predispose to specific symptoms through familiarity with them and their emotional associations as an idiom of disability or distress. High emotional arousal conduces to rapid associative learning and symptom consolidation. Also, attractor selection through exposure to internal or external cues may become more automatic and less open to conscious influence or awareness with the passage of time. The hypothesis here is that neuroadaptational responses stabilize the attractor through associative learning, lowering its threshold for activation relative to alternative responses to comparable stimulus conditions. In turn this is also likely to underpin the propensity for functional symptoms to relapse under conditions of emotional arousal or other relevant cues in relapsing-remitting symptom patterns or during recovery from chronic symptoms. Neuroadaptational changes do not exclude individual variation in the degree of potential conscious influence over functional symptoms given the range of higher cognitive processes that may influence attractor selection at intermediate levels underpinning a given functional symptom. These might include memories or feelings associatively linked to attractors generating the symptom which are potential targets of psychological interventions [2].

To summarize, brain activity at intermediate association cortical levels of the processing hierarchy may be similar in suggested effects and functional symptoms, but upstream regulatory processes may differ ranging from high levels of cognitive control of representations held in working memory for suggested effects compared to more cue-driven, automatic, unconscious and consolidated processes in functional symptoms.

## 6. Comparing different models of top-down modulation applied to suggested effects and functional symptoms

We have considered how three models of cortical function can be applied to the explanation of suggested effects and functional symptoms—HPC, top-down cortical feedback and signal routing and attractor dynamics. All three theories share important features in common. They all explain cognition in terms of the notion that the brain is organized as a graded anatomical hierarchy with bidirectional influences between levels. They allow that specific representations privileged by heightened attention (whether by cognitive control or automatic processes) can influence signal processing in the cortical hierarchy to generate suggested effects and functional symptoms. The cognitive effects of high-level representations occur through an increase in top-down signals relative to bottom-up signals across the cortical hierarchy. The high-level representations are variously described as priors in generative models (HPC), representations of context and goals (Zagha), or high-level cortical attractor states (Rolls). All three approaches posit the propagation of top-down signals resulting in enhanced activation of selected neuronal populations in intermediate levels of the cortical hierarchy, which either alter the interpretation of bottom-up signals or radically misrepresent the presence or absence of bottom-up signals to higher levels (as for example in the case of suggested or functional pain or analgesia respectively). All emphasize that network firing patterns evolve to minimize energy, contributing to stable network activity in low energy states.

There are, however, important differences between the theories. HPC applies Bayes' theory of conditional probabilities to a predictive coding model of brain function involving belief updating via prediction error reconciliation, whereas the signal routing and attractor dynamic approaches do not. The signal routing model does, however, use Mumford's non-Bayesian predictive coding model to account for interactions between higher and lower sensory cortices to generate sensory perception, and a conceptually related forward model account of relations between motor and sensory processing to distinguish voluntary and involuntary movement [19]. Attractor dynamic approaches (as developed by Rolls *et al.*) point to physiological constraints which may limit the implementation of HPC based on belief updating by prediction error reconciliation [25]. In their approach attractor states are established by a combination of positive feedback via recurrent collateral connections and competitive inhibition of alternative attractors implemented by inhibitory interneurons. Rather than strong top-down predictions driving prediction error reconciliation, attractor approaches emphasize discrete processing within successive levels of the cortical hierarchy, strong forward transmission of processed information, and generally weak feedback influences between levels which bias competition between neuronal populations. Nevertheless, accentuation of top-down signals via backprojections is employed in attractor dynamic models of cross-modal perceptual illusions, schizophrenia and OCD (and extended here by analogy to suggested effects and functional symptoms).

A key question concerns, then, whether top-down influences between cortical levels bias competition between neuronal populations (attractors) or bias inference (prediction error reconciliation). Equally, some explanatory constructs may be comparable. The Bayesian term ‘prior’ is used to describe what would otherwise be termed an attractor in Roll's framework without a wider application of Bayesian modelling [25]. In this case, an attractor can be understood as a way in which a prior can be neurally instantiated [80,82]. Zagha's notion of high-level representation of contexts and goals could be understood in terms of priors or attractors. Equally, terms such as ‘precision’ or ‘weight’ in HPC can be applied with modifications to attractor dynamics to represent increases in strength of top-down signals via backprojections. The relationship between differing models can partly be addressed by formal computational modelling of their similarities and differences [83], and their respective fit with ongoing studies of how feedforward and feedback processes are implemented in neuronal circuitry [84] and brain systems [85,86].

Future research employing explicit computational modelling of suggested effects and functional symptoms using HPC and attractor dynamics can also identify potential similarities and differences between the respective phenomena, as well as generate hypotheses about brain mechanisms that can potentially be tested with brain measurement techniques. In this respect future fMRI studies should employ effective connectivity methods in large well-characterized samples to improve understanding of causal relations between regions and networks implicated in suggested effects and functional symptoms. Connectivity and modelling studies should help to identify the key axes and levels in the cortical hierarchy which differentially constrain suggested and functional symptoms. These analyses can be interpreted in terms of cortical source, target and function in accordance with Zagha's proposed anatomical framework for examining modulatory relationships across the cortical hierarchy [19]. They should also help identify similarities and differences in mechanisms of suggested effects and functional symptoms. The use of phenomenological interview methods can also be employed to better understand the range of experiential correlates associated with suggested effects and functional symptoms. Integrating phenomenological methods with brain imaging can potentially inform understanding not only of synchronic variation in the experiential correlates of suggested effects and functional symptoms, but also how they change with symptom chronicity and/or treatment. Such methods can also be linked to the investigation of associated neuroadaptational changes. Phenomenological research can inform understanding of differences in capacities for self-regulation of mental and psychophysiological states, and how this relates to symptom induction and resolution. A related question concerns how mechanisms of suggested effects and functional symptoms can be embedded within a better understanding of how they are influenced by individual variation in mental states and accompanying brain dynamics in distributed networks.

## 7. Combining suggestion and virtual reality in the treatment of functional neurological symptoms

The concept of the cortical hierarchy provides an anatomical framework for understanding how high-level representations such as beliefs, expectations and imagery influence brain function by ‘top-down’ effects, and VR influences brain function by simulating the ‘bottom-up’ effects of sensory input to sensory processing streams. This has implications for treating functional neurological symptoms.

Considered in isolation, treatment of functional symptoms by suggestion is typically applied within the context of other treatment approaches, such as cognitive-behavioural, rehabilitative or psychodynamic therapy. Suggestions are generally symptom-focused (designed to resolve a symptom) or exploratory (using methods such as revivification or age regression to explore experiences associated with symptom onset) [2]. Treatments have predominantly focused on motor symptoms, sensory symptoms and dissociative seizures [2].

VR has a range of applications for treating functional symptoms. To date published studies have focused on the investigation and/or treatment of functional motor disorder, persistent postural perceptual dizziness (PPPD) and dissociative amnesia [4]. Interventions for motor and cognitive FND have employed methods including manipulation of sensory feedback from limb movement, exposure therapy based on FND triggers, or maze walking to investigate egocentric spatial learning in dissociative disorders. VR interventions for PPPD have mainly used VR simulations eliciting movement in different environments and/or reproduced visual motion cues eliciting symptoms.

There are several ways in which suggestion and VR can interact across the cortical hierarchy to treat functional symptoms. The sense of presence in VR is modulated by suggestibility trait [5]. This implies that top-down influences relating to suggestive components of the VR context and stimuli contribute to the experience of VR in addition to sensory input.

It is presently unknown, however, to what extent the effect of suggestibility trait on the sense of presence in VR is influenced by the verisimilitude of VR effects in any given case. The study demonstrating the modulation of presence during VR employed relatively cartoon-like visual stimuli [5]. Had more realistic stimuli been employed it is possible that the sense of presence may have been less reliant on trait suggestibility. This could be tested experimentally by varying the verisimilitude of VR stimuli while measuring presence in a sample of individuals with a range of suggestibility scores. This would allow the hypothesis to be tested that the association between suggestibility score and presence weakens as VR verisimilitude increases. Combining such a study with neuroimaging would also allow investigation of the changes in network activation and connectivity which mediate the top-down modulatory effects of suggestibility on presence for a given VR stimulus set. In turn this would help to resolve the question of the respective contributions of bottom-up and top-down processes on VR experience.

Nevertheless, even if the association between presence and suggestibility is influenced by the verisimilitude of the VR environment, it may still be the case that suggestibility trait additionally influences therapeutic response to a specific VR intervention. Specifically, any effects of suggestibility trait on response to VR treatments may differ with the type of intervention. For example, more individuals ‘pass’ some suggestions (such as arm rigidity) than others (such as post-hypnotic amnesia) [42]. By analogy, some VR interventions may be easier to respond to than others, with suggestibility trait influencing the likelihood of responding for a given individual. This can be tested by including measures of suggestibility in future treatment trials of VR interventions for FND.

Specific, targeted suggestions could also in principle be used to augment the effectiveness of symptom-focused VR, although to date there are no published intervention studies comparing VR interventions for FND with and without augmentation by suggestion [4].

An alternative approach is to use VR to increase response to suggestions. This could be achieved, for example, by deepening relaxation and attentional immersion in suggestions, or exploring the potential for combining verbal with non-verbal suggestive visual and auditory imagery. One such approach has been VR hypnosis, which combines immersion in VR with suggestions to modulate pain perception [87]. The advantage of this approach is to focus research on using VR to augment response to suggestion across the entire population (including those currently classed as low to moderately hypnotically responsive). This is a potentially efficient approach with rapid application given the relative ease with which targeted suggestions to treat functional symptoms can be designed and adjusted in relation to symptom presentation and response [2]. Treatment studies can also be combined with brain measurement and computational modelling to investigate the neural basis for the interaction between suggestion and VR, and mechanisms of change associated with treatment.

In conclusion, in this Opinion piece we have considered how the concept of the cortical hierarchy as the site of top-down and bottom-up effects on cognition and brain function provides a framework for understanding functional neurological symptoms, suggested effects, VR and their interactions.

## Data Availability

This article has no additional data.

## Theme

One contribution of 12 to a discussion meeting issue ‘Digital healthcare for the management of functional neurological disorders’.
